# Genetic Structure and Relationships among Wild and Cultivated Grapevines from Central Europe and Part of the Western Balkan Peninsula

**DOI:** 10.3390/genes11090962

**Published:** 2020-08-20

**Authors:** Goran Zdunić, Katarina Lukšić, Zora Annamaria Nagy, Ana Mucalo, Katarina Hančević, Tomislav Radić, Lukrecija Butorac, Gizella Gyorffyne Jahnke, Erzsebet Kiss, Gloria Ledesma-Krist, Marjana Regvar, Matevž Likar, Andrej Piltaver, Maja Žulj Mihaljević, Edi Maletić, Ivan Pejić, Marion Werling, Erika Maul

**Affiliations:** 1 Institute for Adriatic Crops and Karst Reclamation, Put Duilova 11, 21000 Split, Croatia; katarina.luksic@krs.hr (K.L.); ana.mucalo@krs.hr (A.M.); katarina.hancevic@krs.hr (K.H.); tomislav.radic@krs.hr (T.R.); lukrecija.butorac@krs.hr (L.B.); 2 National Agricultural Research and Innovation Center, Research Institute for Viticulture and Enology, Romai. St. 181, 8261 Badacsonytomaj, Hungary; nagy.zora@szbki.naik.hu (Z.A.N.); jahnke.gizella@szbki.naik.hu (G.G.J.); 3 Szent Istvan University Institute of Genetics and Biotechnology, Páter Károly u. 1, 2100 Gödöllő, Hungary; kiss.erzsebet@mkk.szie.hu; 4 Institut für Geographie und Geoökologie–Abteilung Aueninstitut, Karlsruher Institut für Technologie, Josefstr. 1, 76437 Rastatt, Germany; g.ledesma@t-online.de (G.L.-K.); marion.werling@kit.edu (M.W.); 5 Department of Biology, Biotechnical Faculty, University of Ljubljana, Večna pot 111, 1000 Ljubljana, Slovenia; marjana.regvar@bf.uni-lj.si (M.R.); matevz.likar@bf.uni-lj.si (M.L.); 6 Institute for the Systematics of higher Fungi, Velika vas 17, Slo-1262 Dol pri Ljubljani, Slovenia; anpiltaver@gmail.com; 7 Department of Plant Breeding, Genetics and Biometrics, Faculty of Agriculture, University of Zagreb, Svetošimunska 25, 10000 Zagreb, Croatia; mzulj@agr.hr (M.Ž.M.); ipejic@agr.hr (I.P.); 8 Department of Viticulture and Enology, Faculty of Agriculture, University of Zagreb, Svetošimunska 25, 10000 Zagreb, Croatia; emaletic@agr.hr; 9 Centre of Excellence for Biodiversity and Molecular Plant Breeding (CoE CroP-BioDiv), Faculty of Agriculture, University of Zagreb, Svetošimunska 25, 10000 Zagreb, Croatia; 10 Julius Kühn-Institut, Federal Research Centre for Cultivated Plants, Institute for Grapevine Breeding Geilweilerhof, 76833 Siebeldingen, Germany; erika.maul@julius-kuehn.de

**Keywords:** *V. v.* subsp. *vinifera*, *V. v.* subsp. *sylvestris*, microsatellite, genetic structure, gene flow, wild grape

## Abstract

The genetic diversity and relationship between wild (*Vitis vinifera* L. subsp. *sylvestris* (Gmel.) Hegi and cultivated (*V. vinifera* L. subsp. *vinifera*) grapevine in the western Balkan region and Central Europe have not been studied together previously, although this area has a rich viticultural past. Here, we studied wild grapevine populations sampled from their natural habitats in several countries of the western Balkan region and Central Europe. Their genetic diversity and structure were compared to cultivars that are traditionally in use in this region. A sample set of 243 accessions was genotyped at 20 nuclear microsatellite loci, including 167 *sylvestris* and 76 diverse *vinifera* cultivars. The genetic diversity of the wild grapevines was lower than that of cultivars by all genetic parameters. Both hierarchical and nonhierarchical clustering methods differentiated two main groups, indicating clear separation between wild and cultivated vines but also revealed clear gene flow between the cultivated and wild gene pools through overlaps and admixed ancestry values in the graphs. There was greater affinity to the wild grapes in Central European cultivars than in Balkan cultivars. Fine arrangement of the structure among cultivated grapevines showed differentiation among Central European and Balkan cultivars. These results confirm the divergence of wild grapes from *vinifera* and highlight the “crossroad” role of the western Balkan peninsula in the broader context of European viticulture.

## 1. Introduction

Wild grape (*V. v.* subsp. *sylvestris*) is a close relative of the cultivated grapevine (*V. v.* subsp. *vinifera*) and the only endemic taxon within the *Vitaceae* family in Europe [1]. Research on wild grape intensified at the begining of the 21st century concurrenty with improvement in molecular identification methods, with the primary goals of conserving its biodiversity, clarifying its taxonomic status and identifying traits of interest for grapevine breeding. Today, populations of *V. sylvestris* are endangered and fragmented, in some cases down to <10 individuals [2] or almost completely devastated [3,4,5]. The natural habitats and populations of wild grapes stretch from the Atlantic coast to the western Himalayas and from the Rhine valley to Tunisia [6,7], coexisting with cultivated varieties [1]. In some European countries, *V. sylvestris* is an officially protected species (Spain, France, Germany, and Hungary), while in Croatia, it has “least critical (LC)” status according to the Flora Croatica Database [8].

One of the most important differentiating traits between cultivated and wild grapevine is flower morphology [7,9]. Wild grapes have dioecious male and female plants, while cultivated varieties are mostly hermaphrodites. Dioecism in wild grapes is mentioned in ancient literature from the first century B.C. referring to grapevines with poor yields [10]. That wild grape, as the proposed ancestor of cultivated grape, underwent the dramatic change from dioecy to hermaphroditism is a primary hypothesis regarding grapevine domestication, and one of the most debated [11,12]. During domestication, it is hypothesized that the change from dioecious wild plants to hermaphrodite cultivated plants ensured greater yield and more stable, regular production [13].

Genetic relationships between wild and cultivated grapevines from Central Asia, the Mediterranean and Western Europe indicate considerable gene-flow and complicate differentiation of the two subspecies [14]. Admixed ancestry has been documented in most of research comparing wild and cultivated grapevines [14,15,16]. Gene flow evolutionary happened from wild ancestor to cultivated grapes but it is also possible from cultivated to wild grapevines, forming admixed individuals [17]. These two subspecies are reproductively compatible even though gene flow between them is slow (4–26%). It is linked to different flowering times in *sylvestris* and *vinifera*, and to geographic isolation of wild habitats [17,18,19]. Wild and cultivated grapevines form a genetic and taxonomic continuum, resulting in spontaneous hybrids [16]. Cultivated grapevine has greater overall genetic diversity and heterozygosity than wild [14,16,20] due to the fact that, during domestication, a large diversity was preferred (e.g., berry shape, aroma, yield) which ultimately favors heterozygosity. The grapevine was distributed very early following ancient trade routes that contributed to the development of a large number of varieties. There is evidence of first- and second-generation crosses between wild and cultivated individuals within the wild habitats [18]. Cultivated varieties clustered together with wild grapes from the same area in Georgia [14]. There was introgression of Italian wild grapes in the genome of some Italian cultivated varieties, in particular some local Tuscan varieties [20]. The relatively isolated Sicilian pool of wild populations was closely related to the local cultivated germplasm [9]. Clustering with local varieties was also observed in Portugal [16]. Around 70% of Iberian varieties are related to its *sylvestris* populations [10].

Grapevine classification according to Negrul [21] has been confirmed in many genetic studies [22,23,24], with grape varieties being classified into the following three main groups of proles: *pontica*, *orientalis*, and *occidentalis*. There are lower sub-classification levels for pontica ecotypes—georgica and balkanica.

Research on the presence and diversity of natural populations of wild grape in Croatia and Bosnia and Herzegovina is relatively recent [25]. Hungarian and German *V. sylvestris* populations were described earlier [3,26,27]. Neighboring countries such as Croatia, Bosnia and Herzegovina, Hungary, and Slovenia have had inter-related viticulture since ancient times [28,29], but there is very little data about genetic relationships among wild and cultivated grapevines within this region, or its connection with Western European germplasm. There are historical evidences that exchange of plant materials among distant European winegrowing regions was common. For example, the variety “Heunisch Weiss” (syn. “Gouais blanc”) was most probably introduced to Germany and northwestern France from Eastern Europe [30]. “Heunisch Weiss” is a progenitor of many well-known European grapevine varieties such as Chardonnay and Riesling [31]. Other traditional cultivated varieties from Central Europe such as Traminer share close genetic similarity with *V. sylvestris* [32]. In Germany, it is most likely that selection of many natural seedlings from crosses between wild and cultivated grapes occurred in the Rhine floodplains, where these two subspecies coexisted together for a long time. There is also a presumption based on investigation of the Danubian wild grape population in Austria, the largest wild grape population in Europe, that it is the bastion of the former metapopulation [15]. Relatively high genetic diversity among true wild grapes in the Danubian populations suggested that the Balkan area is one of migrating populations, which contributed to the overall grapevine diversity of the Austrian populations [15]. The Danube river originates in Germany (Baden-Württemberg), passes through 10 different countries, and drains into the Black Sea. The Danube basin is one of the earliest human-settled areas in Europe.

The grapevine gene pools from Middle and Eastern Europe provide valuable insight into the broader context of this historically important area. The present dataset includes SSR genotyping data for 49 *sylvestris* samples from Croatia and 49 cultivated accessions that were previously described [25]. In Germany, more than 80 individuals are thriving in the most northern population of V. *sylvestris* on the island of Ketsch in the Rhine river [33]. The present study characterizes this genetic diversity from the Western Balkan Peninsula and Germany, including Hungary, to analyse its genetic structure and relationships among wild and cultivated grapes. These results will clarify grape taxonomy and aid grapevine breeders.

## 2. Materials and Methods

### 2.1. Plant Material

**Figure 1:**
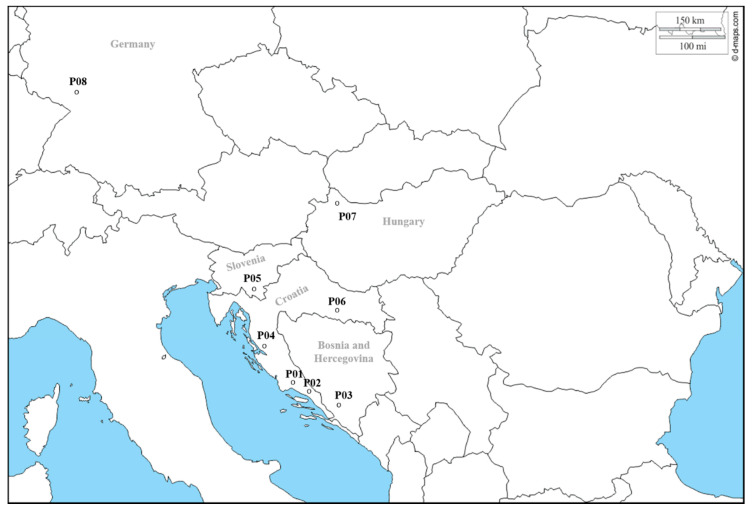
Geographic distribution of the eight sampled *V. v.* subsp. *sylvestris* populations.

The present study is based on 243 non-redundant wild and cultivated grapevine genotypes from Croatia, Bosnia and Herzegovina, Slovenia, Hungary, and Germany (Supplemental Table S1). *V. sylvestris* samples were collected in their natural habitat: wet areas near rivers, lakes and other water reservoirs. Each *sylvestris* candidate was analyzed ampelographically and only those that met the basic dioecious phenotypic profile of wild grapevines were subjected to further genetic analysis. *V. sylvestris* indivuduals were sampled at their natural habitats on the eight different locations: Croatia (P01, P02, P04 and P06), Bosnia and Hercegovina (P03), Slovenia (P05), Hungary (P07), and Germany (P08) (Figure 1).

For analysis of genetic structure and differentiation between wild and cultivated grapevines in these regions, seventy-six *V. v.* subsp. *vinifera* varieties from the corresponding countries were included. According to historic records, summarized in the *Vitis* International Variety Catalogue database (www.vivc.de) [34], wide genetic variability was gathered based on the presumed geographic origin of each variety: Hungary (*n* = 27), Germany (*n* = 16), Croatia (*n* = 17), France (*n* = 4), Bosnia and Hercegovina (*n* = 2), Italy (*n* = 1), Switzerland (*n* = 1), Austria (*n* = 1), Montenegro (*n* = 1), Slovenia (*n* = 1), Balkan (*n* = 1), and unknown (*n* = 4).

### 2.2. DNA Extraction and Microsatellite Analysis

DNA was extracted from young leaves. Total genomic DNA was extracted using the NucleoSpin Plant II kit (Macherey-Nagel, Düren, Germany). The extracted DNA was quantified and used at a working DNA concentration of 1 ng/μL. Twenty microsatellite loci were analyzed to study the genetic diversity of the samples: VMC1B11 [35]; VMC4F3.1 [36]; VrZAG62 and VrZAG79 [37]; VVIB01, VVIN16, VVIN73, VVIP31, VVIP60, VVIQ52, VVIV37, and VVIV67 [38]; VVMD5, VVMD7, VVMD21, VVMD24, VVMD25, VVMD27, and VVMD28 [39,40]; and VVS2 [41]. All forward primers were 5’ end-labeled with fluorescent dyes (FAM, HEX, TAMRA, or ROX). The combinations of microsatellite loci (multiplexes) were optimized at the Julius Kühn-Institut laboratory; using different labels and diverse fragment lengths allowed multiplexing of the polymerase chain reactions (PCR) with up to four markers. Characteristics of markers and PCR multiplex combinations are presented (Supplemental Table S2).

The KAPA Fast Multiplex PCR Kit (2x) (Kapa Biosystems, Wilmington, MA, USA) was used to set up reaction mixtures containing master mix, 100 pmol of each primer, and ~ 1 ng template DNA. Amplification was performed in ABI 9700 thermal cyclers (Applied Biosystems, Foster City, CA, USA) using the following program: three min initial denaturation at 95 °C, followed by 30 cycles of denaturation at 95 °C (15 s), annealing at 60 °C (30 s), and extension at 72 °C (30 s). A final extension was performed at 72 °C for seven min. DNA of two certified cultivars from Julius Kühn-Institut laboratory, “Muscat á petits grains” and “Cabernet franc,” were amplified and used as references to standardize the allele calls.

Amplified products were resolved using capillary electrophoresis on an ABI 3130xl Genetic Analyzer (Applied Biosystems, Foster City, CA, USA) using GeneScan-LIZ 500 as an internal standard. Peaks were identified by size and height with GeneMapper 5.0 software (Applied Biosystems, Foster City, CA, USA).

### 2.3. Data Analysis

Different measures of genetic variability among 243 unique genotypes at 20 SSR loci were calculated. The number of alleles per locus (Na), number of effective alleles (Ne), private alleles, observed (Ho) and expected (He) heterozygosity, and Fixation index (F) were calculated for each locus over both wild populations and cultivated grapevines using GenAlEx 6.5 [42].

Genetic relationships among accessions were assessed by distance-based cluster analysis using the neighbor-joining method (NJ) as implemented in the MEGA 6.0 software [43] using the codominant genotypic distances between all pairwise combinations calculated by the GenAlEx 6.5 [42]. The bootstrap interior branch test implemented in MEGA 6.0 software [43] was used to test the reliability of each interior branch on tree. Principal coordinate analysis (PCA) was used to display genetic divergence among samples using codominant genotypic distances computed in GenAlEx 6.5 [42].

The genetic differentiation between populations was carried using pairwise F_ST_ and pairwise Nei’s genetic distance for each pairwise combination of populations in GenAlEx software [42].

Bayesian model-based cluster analysis executed in STRUCTURE [44] was applied to infer the genetic structure of the investigated accessions and to assign individuals to populations. The STRUCTURE configuration was set to ignore population information and use an admixture model with correlated allele frequencies. The degree of admixture α was inferred from the data. α is close to zero when most individuals are from one population or another, while α is >1 when most individuals are admixed. The allele frequency parameter (Lambda) was set to one as suggested in the STRUCTURE manual. Various numbers of putative populations (K) were tested, ranging from 1 to 10. Burning time and replication number were set to 100,000 and 100,000, respectively, in each independent run with 10 iterations. The choice of the most likely number of clusters (best K) was evaluated in accordance with the ad hoc statistic delta K as described [45] using Structure Harvester [46]. Structure bar plot was visualized by running the clumpp file (K = 2) obtained by Structure Harvester, in Structure Plot v 2.0 [47]. Structure ancestry Q values for each analysed individual are presented in Supplemental Table S3, with the highlighted values of Q > 0.75 representing reliable ancestry assignment to its own cluster.

## 3. Results

The genetic indices of the 167 wild and 76 cultivated accessions at 20 nuclear SSR loci were calculated (Table 1) after the SSR profiles of all 243 analyzed samples were determined (Table S3). The total mean number of alleles (Na) was 11 and ranged from four (VVIQ52) to 19 (VMC4F3.1). The number of effective alleles (Ne) ranged from 1.957 (VVIQ52) to 6.512 (VVIV67), with an overall mean of 3.658. Observed and expected heterozygosity was least for VVIQ52; the largest Ho was for VVMD7 while the largest He was for VVIV67. VVMD7 is reported as the most informative marker in *V. sylvestris* [48]. The expected heterozygosity (He) values varied between 0.489 (VVIQ52) and 0.846 (VVIV67), with an average of 0.698. Overall mean observed heterozigosity was lower than the expected heterozygosity. The observed heterozygosity (Ho) values ranged from 0.364 (VVIQ52) to 0.755 (VVMD7), with an overall mean of 0.578. Fixation index (F), a measure of reduction in heterozygotes and thus an indicator of greater inbreeding, was least for VVMD7 (0.047) and greatest for VVMD21 (0.340). The mean F was 0.174.

Genetic diversity indices at the population level show that the number of alleles per locus of cultivated grapevines was greater (8.600) than in wild grapevines, where Na values ranged from 3.100 (Hungarian wild set = P07) to 5.300 (Bosnian wild set = P03) (Table 2). The overall Ne value in the dataset was 2.739. Cultivated varieties had the greatest (4.471) Ne values, while P07 had the lowest value (2.000) and the Croatian P04, the highest (2.796) among the wild populations. The Ho values in *vinifera* (0.759) were greater than that of any wild population, which ranged from 0.437 in P08 to 0.593 in P06. The He values were greater for *vinifera* grapevines than for *sylvestris*. Fixation index values were positive for all analysed groups except for cultivated varieties, indicating heterozygosity deficiency or greater inbreeding within wild populations. The group of cultivars had a negative F, indicating more heterozygosity. The F values within *sylvestris* were least for P07 (0.013) and greatest for P05 (0.146)

Genetic distance among the eight *sylvestris* populations and the group of cultivated varieties was estimated using pairwise Fst values and Nei’s genetic distance. (Table 3). The Nei’s genetic distance values ranged from 0.039 (Croatia-Bosnia *sylvestris*, P01–P03) to 0.625 (cultivated group–Germany *sylvestris* P08). The F_ST_ values ranged from 0.067 for the Croatian *sylvestris* (P04) and Slovenian *sylvestris* (P05) up to 0.163 for the *sylvestris* from Croatia (P01 and P02) but was greatest for *sylvestris* P01-cultivated samples (0.187).

Fifty-seven private alleles (PA) were found at 19 out of 20 SSR markers in the studied set (Supplemental Table S4). In *sylvestris*, PA were observed at 15 out of 20 SSR. The number of PA was lower in *sylvestris* (22) than in the cultivated *vinifera* (35), but not drastically so. In cultivated grapevines, PA were found at 14 out of 20 SSR loci. In this study, 22 *sylvestris* PA were observed in seven out of eight populations. The greatest number of private alleles was identified in P06 (6) followed by P05 (5), P03 (4), and P08 (3), all populations from different countries.

**Figure 2:**
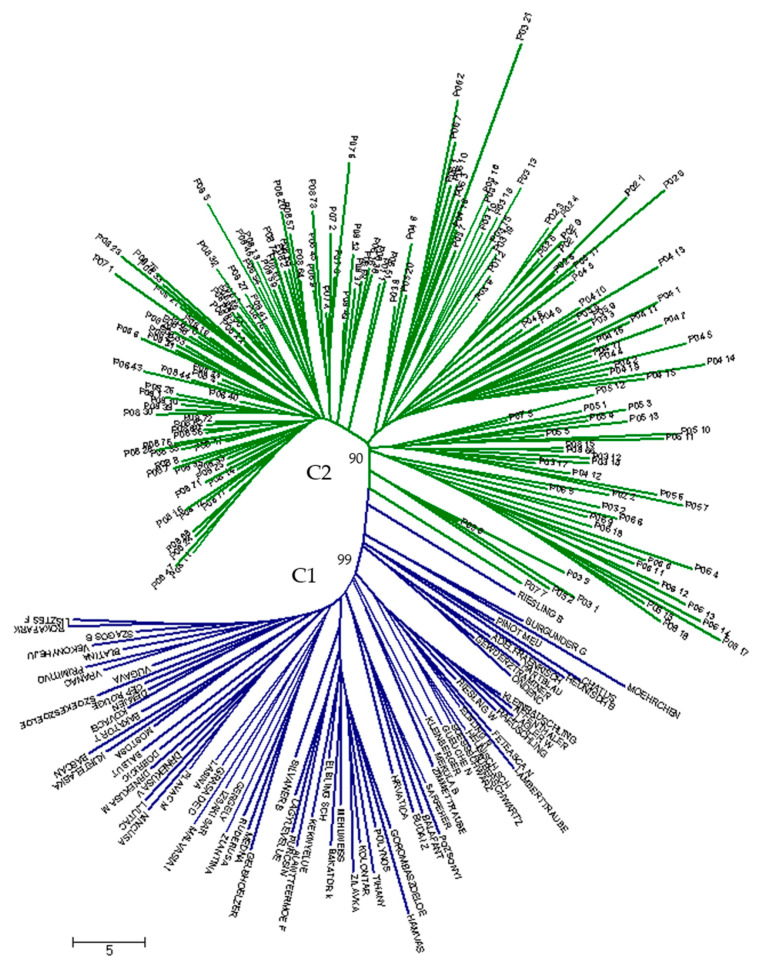
Neighbor-joining dendrogram showing genetic relationship among 243 wild and cultivated grapevine accessions based on 20 SSR loci. Cultivated samples clustered in cluster C1 (blue color), while wild samples clustered in cluster C2 (green color) with bootstrap support value ≥90%.

The neighbor-joining (NJ) cluster analysis showed clear differentiation between the *vinifera* and *sylvestris* subspecies, which formed two main clusters, one of which contained cultivated grapes (C1), and other (C2), the wild grapes from Germany, Hungary, Slovenia, Croatia, and Bosnia and Herzegovina (Figure 2). One set of Western European wine cultivars (“Riesling blau,” “Burgunder gross,” “Moehrchen,” “Pinot meunier,” “Chatus,” “Heunisch blau,” “Adelfraenkisch,” “Hartblau,” “Gewuerztraminer,” and “Ondenc”) showed affinity for the wild grapes, forming the basal group to the rest of the cultivated grape. There are also a few *sylvestris* individuals from various wild populations (P07_7, P05_2, P03_1, P03_5, and P03_6) that are closely allied with the cultivars. Wild accessions tend to cluster together with individuals from the same population due to high within-population genetic similary. The geographic clustering can be seen in the *vinifera* set originating from at least 10 different countries tending to differentiate between Western and Eeastern European cultivars.

**Figure 3:**
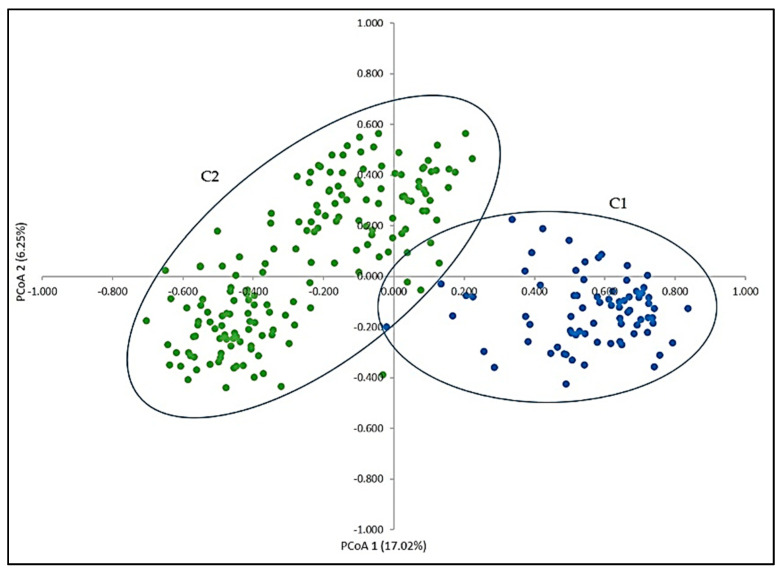
Principal coordinate analysis (PCA) of the 243 wild and cultivated samples represented by two axes using a covariance matrix of 20 SSR loci. Cultivated samples are represented in group C1 (blue) and wild samples are in group C2 (green).

The structure and the correlations among individuals were analysed using PCooA based on the genetic distance matrix of 20 SSRs. PCooA projections were plotted in a two-dimensional scatter plot (Figure 3). The PCooA maximizes the linear correlation between distance in the distance matrix and distance in the 2-D space, giving insight into the relative relationships among accessions. Projection of the first two principal axes accounted for 23.27% of the total molecular variation. The first dimension (PCooA1) explained 17.02% while the second dimension (PCooA2) explained 6.25% of the total variation in the set. Genetically similar individuals were highly correlated and clustered together, forming two groups (cultivars with cultivars-C1 and *sylvestris* with *sylvestris*-C2). Despite the strict pre-analysis selection of presumed pure *sylvestris*, PCooA revealed slight overlap between C1 (*vinifera*) and C2 (*sylvestris*), indicating gene flow from *vinifera* to *sylvestris* and vice versa, even though the *vinifera* set was half the size of the wild set.

**Figure 4:**
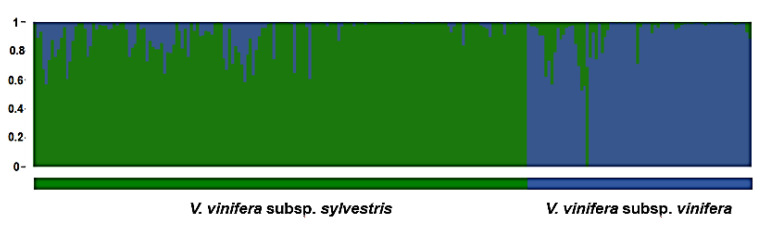
Graphic presentation of the population structure of 243 grapevine accessions. Each accession is represented by a single vertical bar divided into K color segments representing its proportions in the two inferred genetic clusters using STRUCTURE software. Wild accessions grouped into a population represented by green, while cultivated accessions grouped into a population represented by blue.

Non-hierarchical horizontal clustering with the Structure software assigned the 243 specimens (167 *V. sylvestris* and 76 *V. vinifera*) into two clusters: *sylvestris* and *vinifera* (Figure 4) based on the optimal number of clusters (K) calculated as described [46] (Supplemental Figure S1). Both groups were clearly separate, but also showed some admixture, corresponding to the overlap observed in the NJ and PCooA analysis. The accessions with Q < 75 were considered accessions with admixed origin. All populations except P05 (Slovenia) showed admixed *sylvestris/vinifera* ancestry. Among wild populations, the most accessions of admixed ancestry were observed in Croatian populations P06 (Psunj) and P02 (Imotski). Populations P07 (Hungary) and P08 (Germany) had only one such accession. The greatest percentage of admixed genomes among cultivated *V. vinifera* was observed in Western wine cultivars: “Pinot meunier,” “Burgunder gross,” “Moehrchen,” “Suessschwarz,” “Adelfraenkisch,” “Gewuerztraminer,” “Riesling blau,” “Chatus,” and “Ondenc.” Other cultivars were all of *V. vinifera* ancestry with Q > 75 (Supplemental Table S5). Structure showed similar clustering to NJ and PCooA. However, NJ and Structure were more subtle, providing more detailed insight into the genetic structure among individuals than the distance-based PCooA. Detection of intermediate position between the groups (overlaps) on the graph is neverthless facilitated by PCooA, but the multiple dimensions to show the relationship between genotypes provided by clustering methods.

## 4. Disscussion

The western Balkans and Central Europe were primary transit routes of grapevine varieties on their way from the east to the west [23,49]. The main objective of this study was to investigate genetic diversity, relationships and structure between wild and putative autochthonous varieties in this area to find whether their common territorial and historical ground is reflected in viticulture, and to describe the wild gene pool in relation to the cultivated pool of this area.

Genetic diversity in the studied set of individuals, expressed through the expected and observed heterozygosities of SSR markers, was greater in *vinifera* than in *sylvestris,* as seen in previous studies [9,16,18,20]. Among *sylvestris* populations, the greatest heterozygosity was observed in P06 (Croatia) and the lowest for P08 (Germany), indicating heterozygosity decreased from east to west. The east-west pattern of decreasing diversity was documented in other studies [4,24]. It is presumed that *sylvestris* populations in the Rhine floodplains followed a south-north migration route after glaciation retreated, being thus more related to the Southwestern European gene pool. Decreasing heterozygosity was confirmed by the fixation index, in general highlighting inbreeding in all *sylvestris* populations, while the cultivated set had a negative fixation index, showing greater genetic diversity than *sylvestris*. However, this situation is not expected in *sylvestris,* as the presumed ancestor of cultivated grapes. The F_ST_ statistics showed low differentiation between *vinifera* and *sylvestris*, in agreement with previous results [14,49]. It was greater between *sylvestris*–*sylvestris* (Croatia–Slovenia), but even greater between *sylvestris* (Croatia, P01) and cultivated varieties. Genetic diversity by all measures was reduced in *sylvestris* populations, indicating their isolation, where sibmating leads to inbreeding and loss of alleles and heterozygosity. Private alleles can be useful indicators of gene flow [50] or used as discriminant markers to reliably distinguish *sylvestris* from *vinifera* [23]. In this study, while overall private allelic richness was lower in *sylvestris* than in the cultivated set, there were many private alleles in the *sylvestris* populations from all countries, highlighting the local richness of the wild populations. There was continuous reduction in genetic variability of *Vitis sylvestris* populations, leading to loss of alleles and heterozygosity. In general, perennial fruit crops such as grape are the descendants of spontaneous or controlled crosses between geographically and genetically distant individuals, resulting in new variants carring novel combinations of alleles not find in the wild [51]. Therefore, cultivars contain more genetic variability than the remaining wild grape populations. Unique alleles observed in only one population or individual can be useful in determining to which population a particular individual belongs, as indicators of gene flow [50].

By using the combined *vinifera* –*sylvestris* set, the NJ, PCooA, and STRUCTURE analyses confirmed clear separation of the two groups. Both groups showed high assignment to their own cluster, confirming the representativeness of the studied set. The accessions falling in the transition zone in NJ, the overlapping zone in PCooA or the admixed part in Structure suggest a common gene pool for the two groups. Despite the strict pre-selection of true-to-type *sylvestris* individuals and a cultivated set originating from at least 10 different countries that was only half as large, classification (hierarchical, nonhierarchical) and ordination (PCooA) methods both showed bidirectional gene flow between the *vinifera* and *sylvestris* gene pools.

The NJ cluster analysis was carried out without considering the geographic origin of the samples. However, the cultivated set displays a clear structured arrangement of accessions by ancestry, diversity and putative geographic origin. “Elbling Blau” and eight wine grapes in the first three independent small subclusters are first-degree related to ‘Savagnin’ (specifically, its red mutant ‘Traminer’) and ‘Pinot’. The genetic proximity of “Savagnin”/“Traminer” and “Pinot” to *sylvestris* shown here was reported previously by various authors [1,14,49]. All but two varieties in the subsequent cluster comprising 12 varieties are first-degree related to “Heunisch,” with one variety being first- and four varieties being second-degree related to “Savagnin”/“Traminer.” Genetic composition of “Riesling Weiss,” “Elbling,” and “Raeuschling” and morphology place them close to “Savagnin”/“Traminer.” The presence of alleles of the at least 2000-year-old “Savagnin” [52] in the subgroup possibly explains the position near to the *sylvestris* cluster. Most of these wine grapes, except “Riesling Weiss,” “Pinot,” and “Gewürztraminer,” are minor varieties or were rediscovered in recent times. The subsequent clusters encompass mainly Hungarian varieties, followed by Croatian and Bosnian with some intermixed Hungarian varieties. Clustering far from *sylvestris* and high *vinifera* ancestry values point to a different geographic origin, most likely the Near East [50]. In the wild cluster, the structure is fragmented but follows the geographic pattern of the populations, confirming high similarity among wild populations and their common gene pool. The Q values indicate admixed individuals in all wild populations. The Croatian population from Psunj (P06) had the most admixed individuals (7), suggesting more recent introgression from cultivated grapes, while in other wild populations, such individuals were rarer. Wild accessions from Germany and Hungary were far less likely to be assigned to *vinifera* cluster, while cultivars from Germany were grouped close to the *sylvestris* cluster. “Riesling blau,” the cultivar in the transitional zone in NJ, shared more alleles with *sylvestris* than with the *vinifera* group in this set of samples (Q: *sylvestris* = 0.69, *vinifera*= 0.31). The same pattern of allele sharing from *sylvestris* was seen in some other Western European cultivars in other genetic comparison studies [14,49,50]. When analysed using SSRs, the cultivar “Manseng” clustered with *sylvestris* in a Structure plot [23], but SNP analysis clustered it among *vinifera*. One should take into consideration the limited power of SSR markers to resolve subtle phylogenetical relations among closely related individauls [23,53].

Because of its reduced number of dimensions, PCooA provides less-detailed insight into the genetic structure of the studied set and does not allow conclusions as to what extent some population/individual influenced the other. However, this analysis facilitates detection of an intermediate position between the groups (overlaps). Clustering methods give more detailed information on the genomic nature of the studied set. Both groups showed very high average probabilities of assignment to their own cluster, consistent with being genetically different.

Outlier groupings or diversity was not observed, giving insight into the similarity of the studied wild populations from this area. Greater diversity was observed in the eastern than in the western wild populations, and southern populations were more diverse than the northern ones, so the decrease in diversity can be traced from east to west, and from south to north. Ketch island in Germany was the northernmost population, while Dalmatian populations were most southern. In the Balkans, one study assumed there was a past metapopulation covering the broader area of Danube river [15]. Several populations from different countries belong to this area. All studied *Vitis sylvestris* populations represent small, isolated populations surviving in mountain or floodplain forests in close proximity to rivers. The remaing populations of *Vitis sylvestris* are considered an “endangered species” [15]. Several sites (P02, P04, P07 and P08) belong to protected areas of flora and fauna biodiversity, where no direct human degradation is allowed, but all sites are endagenred due to taxonomic pollution through gene flow from other *Vitis* taxa that escaped from vineyards [15]. Gizdavac (P01) is a highly endangered wild population with only two individuals (female and male) recorded.

This study demonstrated that populations of wild grapes are highly fragmented into small, isolated populations that represent valuable genetic resources. Additional conservation efforts are needed to protect the remaining genetic variation of *V. v.* subsp. *sylvestris* resources.

## 5. Conclusions

Analysis of Western Balkan and Central European *V. vinifera* and *V. sylvestris* populations showed clear distinction between cultivated and wild grapevines. Despite strict selection of pure *sylvestris* and a considerably smaller set of *vinifera* cultivars included in analysis, all statistical and phylogenetic methods showed high assignment of *vinifera* to *vinifera* and *sylvestris* to *sylvestris* clusters, but always with visible overlaps between the groups. Through hierarchical phylogenetical clustering, *vinifera* cultivars were shown to be the source of genetic variation in the studied set. Allelic richness of the wild gene pool from various countries indicated local adaptation of *sylvestris* populations and assignment to its population of origin, confirming a valuable source of genetic variation in the studied populations. Close affinity was seen between the studied *sylvestris* populations and Western European cultivars, suggesting a common genetic origin. In contrast, cultivated grapes from the Balkan area were distant from the *sylvestris* populations in this sample set, suggesting a different geographic and genetic pool of domestication. However, the general questions relating the time and direction of introgression between these two subspecies are still open and could be resolved with a broader sample set and whole-genome data.

## Figures and Tables

**Table 1:** Genetic diversity indices calculated for 243 genotypes, including wild and cultivated accessions.

Locus	N	Na ^a^	Ne	Ho	He	F
Zag62	242	7	2.818	0.488	0.645	0.244
ZAG79	243	11	2.315	0.486	0.568	0.145
VVIV67	234	18	6.512	0.697	0.846	0.177
VVIN16	243	5	2.460	0.506	0.593	0.147
VVIP60	241	12	4.390	0.664	0.772	0.140
VVMD25	239	11	4.720	0.674	0.788	0.145
VVIN73	241	6	2.062	0.465	0.515	0.098
VVMD5	237	10	4.449	0.692	0.775	0.107
VVIB01	235	7	3.130	0.485	0.681	0.287
VVMD24	234	8	4.257	0.654	0.765	0.145
VVMD27	242	8	2.515	0.500	0.602	0.170
VVIQ52	236	4	1.957	0.364	0.489	0.255
VVS2	240	13	4.009	0.608	0.751	0.190
VVIV37	235	11	4.029	0.626	0.752	0.168
VMC4F3.1	236	19	3.754	0.648	0.734	0.116
VMC1B11	240	11	3.590	0.538	0.721	0.255
VVMD21	209	11	2.709	0.416	0.631	0.340
VVMD28	236	14	3.720	0.623	0.731	0.148
VVIP31	243	13	4.941	0.671	0.798	0.159
VVMD7	241	13	4.815	0.755	0.792	0.047
Mean	237.4	11	3.658	0.578	0.698	0.174

^a^ Na: number of different alleles; Ne: number of effective alleles; Ho: observed heterozygosity; He: expected heterozygosity; and F: fixation index.

**Table 2:** Genetic diversity estimates for each analyzed population of wild and cultivated grapevines. Population P01 was excluded due to an insufficient number of individuals (only two) for population genetic indices.

Population		N	Na ^a^	Ne	Ho	He	F
P02	Mean	9.0	3.700	2.583	0.586	0.576	0.016
	SE	0.1	0.263	0.186	0.062	0.028	0.081
P03	Mean	20.6	5.300	2.735	0.576	0.606	0.051
	SE	0.2	0.252	0.170	0.034	0.025	0.035
P04	Mean	18.6	4.850	2.796	0.531	0.572	0.069
	SE	0.2	0.406	0.279	0.046	0.041	0.040
P05	Mean	12.6	4.050	2.513	0.456	0.538	0.146
	SE	0.2	0.294	0.208	0.053	0.045	0.067
P06	Mean	16.9	4.700	2.785	0.593	0.603	0.021
	SE	0.2	0.282	0.201	0.046	0.029	0.054
P07	Mean	7.0	3.100	2.000	0.464	0.444	0.013
	SE	0.0	0.191	0.160	0.059	0.039	0.089
P08	Mean	75.7	4.550	2.034	0.437	0.447	0.020
	SE	1.2	0.328	0.149	0.048	0.045	0.026
Cultivars	Mean	75.2	8.600	4.471	0.759	0.733	-0.038
	SE	0.5	0.682	0.399	0.030	0.029	0.011
Total	Mean	29.4	4.856	2.739	0.550	0.565	0.036
	SE	2.1	0.177	0.098	0.018	0.014	0.020

^a^ Na: number of different alleles; Ne: number of effective alleles; Ho: observed heterozygosity; He: expected heterozygosity; and F: fixation index.

**Table 3:** Estimates of F_ST_ values (below diagonal) and Nei’s unbiased genetic distance (above the diagonal).

	P01	P02	P03	P04	P05	P06	P07	P08	Cultivars
P01	-	0.260	0.039	0.130	0.174	0.286	0.126	0.154	0.604
P02	**0.163**	-	0.181	0.293	0.327	0.299	0.422	0.348	0.482
P03	**0.092**	**0.069**	-	0.101	0.133	0.197	0.179	0.168	0.548
P04	**0.123**	**0.098**	**0.044**	-	0.157	0.227	0.227	0.263	0.554
P05	**0.137**	**0.116**	**0.059**	**0.067**	-	0.179	0.130	0.218	0.600
P06	**0.159**	**0.091**	**0.062**	**0.074**	**0.071**	-	0.242	0.313	0.489
P07	**0.131**	**0.157**	**0.084**	**0.098**	**0.076**	**0.107**	-	0.118	0.551
P08	**0.139**	**0.137**	**0.077**	**0.102**	**0.100**	**0.116**	**0.074**	-	0.625
Cultivars	**0.187**	**0.102**	**0.099**	**0.110**	**0.127**	**0.094**	**0.141**	**0.150**	-

In bold, significant F_ST_ values with *p* ≤ 0.05, calculated over 999 permutations.

## Supplementary Materials

The following are available online at https://www.mdpi.com/2073-4425/11/9/962/s1. Table S1: List of samples used in this study, Table S2: characteristics of 20 SSR markers used in this study, Table S3: SSR profiles of all 243 analyzed samples, Table S4: List of private alleles (and allele frequencies) in a 243-sample set of *V. sylvestris* and *V. vinifera*, Table S5: Q values of 243 samples obtained from Structure software analysis, Figure S1: Bayesian assignment analysis as: (a) a plot of mean likelihood L(K) and variance per K value exploited by STRUCTURE software on a data set of 243 individuals based on 20 SSR loci; (b) The optimal number of K that best fit the data set is two.

## References

[1] De Andrés M.T., Benito A., Pérez-Rivera G., Ocete R., Lopez M.A., Gaforio L., Muñoz G., Cabello F., Martínez Zapater J.M., Arroyo-García R. (2012). Genetic diversity of wild grapevine populations in Spain and their genetic relationships with cultivated grapevines: Genetic diversity of wild grapevine populations from Spain. Mol. Ecol..

[2] Ocete R., Lopez M., Gallardo A., Arnold C. (2008). Comparative analysis of wild and cultivated grapevine (*Vitis vinifera*) in the Basque Region of Spain and France. Agric. Ecosyst. Environ..

[3] Schröder S., Kortekamp A., Heene E., Daumann J., Valea I., Nick P. (2015). Crop wild relatives as genetic resources—The case of the European wild grape. Can. J. Plant. Sci..

[4] Biagini B., Imazio S., Scienza A., Failla O., De Lorenzis G. (2016). Renewal of wild grapevine (*Vitis vinifera* L. subsp. sylvestris (Gmelin) Hegi) populations through sexual pathway: Some Italian case studies. Flora.

[5] Meléndez E., Puras P., García J.L., Cantos M., Gómez-Rodríguez J.A., Íñiguez M., Rodríguez Á., Valle J.M., Arnold C., Ocete C.A. (2016). Evolution of wild and feral vines from the Ega river gallery forest (Basque country and Navarra, Spain) from 1995 to 2015. OENO One.

[6] Biagini B., De Lorenzis G., Imazio S., Failla O., Scienza A. (2014). Italian wild grapevine (*Vitis vinifera* L. subsp. sylvestris) population: Insights into eco-geographical aspects and genetic structure. Tree Genet. Genomes.

[7] Arnold C., Gillet F., Gobat J.M. (1998). Situation de la vigne sauvage *Vitis vinifera* ssp. Silvestris en Europe. Vitis.

[8] FCD-Flora Croatica Database. https://Hirc.Botanic.Hr/Fcd/.

[9] De Michele R., La Bella F., Gristina A.S., Fontana I., Pacifico D., Garfi G., Motisi A., Crucitti D., Abbate L., Carimi F. (2019). Phylogenetic Relationship Among Wild and Cultivated Grapevine in Sicily: A Hotspot in the Middle of the Mediterranean Basin. Front. Plant. Sci..

[10] Ocete C.A., Arroyo R., Lovicu G., Rodríguez-Miranda Á., Valle J.M., Cantos M., Garcia J.L., Lara M., González De Canales F., Llompart J. (2019). An inventory of the relic Eurasian wild grapevine populational nuclei in Huelva province (Andalusia, Spain). Vitis.

[11] Riaz S., Boursiquot J.-M., Dangl G.S., Lacombe T., Laucou V., Tenscher A.C., Walker M. (2013). Identification of mildew resistance in wild and cultivated Central Asian grape germplasm. BMC Plant. Biol..

[12] Ramos M.J.N., Coito J.L., Fino J., Cunha J., Silva H., De Almeida P.G., Costa M.M.R., Amâncio S., Paulo O.S., Rocheta M. (2017). Deep analysis of wild Vitis flower transcriptome reveals unexplored genome regions associated with sex specification. Plant. Mol. Biol..

[13] This P., Lacombe T., Thomas M. (2006). Historical origins and genetic diversity of wine grapes. Trends Genet..

[14] Riaz S., De Lorenzis G., Velasco D., Koehmstedt A., Maghradze D., Bobokashvili Z., Musayev M., Zdunic G., Laucou V., Walker A.M. (2018). Genetic diversity analysis of cultivated and wild grapevine (*Vitis vinifera* L.) accessions around the Mediterranean basin and Central Asia. BMC Plant. Biol..

[15] Arnold C., Bachmann O., Schnitzler A. (2017). Insights into the Vitis complex in the Danube floodplain (Austria). Ecol. Evol..

[16] Cunha J., Ibáñez J., Teixeira-Santos M., Brazão J., Fevereiro P., Martínez-Zapater J.M., Eiras-Dias J.E. (2020). Genetic Relationships Among Portuguese Cultivated and Wild *Vitis vinifera* L. Germplasm. Front. Plant. Sci..

[17] Di Vecchi-Staraz M., Laucou V., Bruno G., Lacombe T., Gerber S., Bourse T., Boselli M., This P. (2009). Low Level of Pollen-Mediated Gene Flow from Cultivated to Wild Grapevine: Consequences for the Evolution of the Endangered Subspecies *Vitis vinifera* L. subsp. silvestris. J. Hered..

[18] Ghaffari S., Hasnaoui N., Zinelabidine L.H., Ferchichi A., Martínez-Zapater J.M., Ibáñez J. (2014). Genetic diversity and parentage of Tunisian wild and cultivated grapevines (*Vitis vinifera* L.) as revealed by single nucleotide polymorphism (SNP) markers. Tree Genet. Genomes.

[19] Lewter J., Worthington M.L., Clark J.R., Varanasi A.V., Nelson L., Owens C.L., Conner P., Gunawan G. (2019). High-density linkage maps and loci for berry color and flower sex in muscadine grape (Vitis rotundifolia). Theor. Appl. Genet..

[20] D’Onofrio C. (2020). Introgression Among Cultivated and Wild Grapevine in Tuscany. Front. Plant. Sci..

[21] Negrul A. (1946). Ampelography of USSR. Orig. Cultiv. Vine Classif..

[22] Arroyo-García R., Ruiz-García L., Bolling L., Ocete R., López M.A., Arnold C., Ergul A., Söylemezo˝Lu G., Uzun H.I., Cabello F. (2006). Multiple origins of cultivated grapevine (*Vitis vinifera* L. ssp. sativa) based on chloroplast DNA polymorphisms: Multiple origins of cultivated grapevine. Mol. Ecol..

[23] Bacilieri R., Lacombe T., Le Cunff L., Di Vecchi-Staraz M., Laucou V., Genna B., Péros J.-P., This P., Boursiquot J.-M. (2013). Genetic structure in cultivated grapevines is linked to geography and human selection. BMC Plant. Biol..

[24] Emanuelli F., Lorenzi S., Grzeskowiak L., Catalano V., Stefanini M., Troggio M., Myles S., Martinez-Zapater J.M., Zyprian E., Moreira F.M. (2013). Genetic diversity and population structure assessed by SSR and SNP markers in a large germplasm collection of grape. BMC Plant. Biol..

[25] Zdunić G., Maul E., Hančević K., Leko M., Butorac L., Mucalo A., Radić T., Šimon S., Budić Leto I., Žulj Mihaljević M. (2017). Genetic Diversity of Wild Grapevine [*Vitis vinifera* L. subsp. sylvestris (Gmel.) Hegi] in the Eastern Adriatic Region. Am. J. Enol. Vitic..

[26] Bartha D., Kevey B., Tiborcz V. (2012). Current and 20th century distributions of Vitis sylvestris in Hungary. Folia Oecol..

[27] Jahnke G., Nagy Z.A., Koltai G., Hajdu E., Májer J. (2016). Preservation and Characterization of Woodland Grape (Vitis vinifera Ssp. Sylvestris GMEL.) Genotypes of the Szigetköz, Hungary. Germplasm: Characteristics, Diversity and Preservation.

[28] Rusjan D., Bubola M., Janjanin D., Užila Z., Radeka S., Poljuha D., Pelengić R., Javornik D., Štajner N. (2015). Ampelographic characterisation of grapevine accessions denominated “Refošk”, “Refosco”, “Teran” and “Terrano” (*Vitis vinifera* L.) from Slovenia, Croatia and Italy. Vitis.

[29] Maletić E., Pejić I., Karoglan Kontić J., Zdunić G., Preiner D., Šimon S., Andabaka Ž., Žulj Mihaljević M., Bubola M., Marković Z. (2015). Ampelographic and genetic characterization of Croatian grapevine varieties. Vitis.

[30] Maul E., Eibach R., Zyprian E., Töpfer R. (2015). The prolific grape variety (*Vitis vinifera* L.) ‘Heunisch Weiss’ (= ‘Gouais blanc’): Bud mutants, “colored” homonyms and further offspring. Vitis.

[31] Lacombe T., Boursiquot J.-M., Laucou V., Di Vecchi-Staraz M., Péros J.-P., This P. (2013). Large-scale parentage analysis in an extended set of grapevine cultivars (*Vitis vinifera* L.). Theor. Appl. Genet..

[32] Regner F., Stadlhuber A., Eisenheld C., Kaserer H. (2000). Considerations about the evolution of grapevine and the role of Traminer. Acta Hortic..

[33] Ledesma-Krist G.M., Schumann F., Maul E. (2014). Die Wildrebenpopulation auf der Rheininsel Ketsch—Eine wertvolle genetische Ressource. DWJ.

[34] Maul E., Sudharma K.N., Ganesch A., Brühl U., Hundemer M., Kecke S., Mahler-Ries A., Marx G., Schreiber T., Walk M. 30 years VIVC—Vitis International Variety Catalogue. Proceedings of the XI International Conference on Grapevine Breeding and Genetics.

[35] Zyprian E., Topfer R. (2005). Development of Microsatellite-Derived Markers for Grapevine Genotyping and Genetic Mapping.

[36] Di Gaspero G., Peterlunger E., Testolin R., Edwards K.J., Cipriani G. (2000). Conservation of microsatellite loci within the genus Vitis. Theor. Appl. Genet..

[37] Sefc K.M., Regner F., Turetschek E., Glössl J., Steinkellner H. (1999). Identification of microsatellite sequences in Vitis riparia and their applicability for genotyping of different Vitis species. Genome.

[38] Merdinoglu D., Butterlin G., Bevilacqua L., Chiquet V., Adam-Blondon A.-F., Decroocq S. (2005). Development and characterization of a large set of microsatellite markers in grapevine (*Vitis vinifera* L.) suitable for multiplex PCR. Mol. Breed..

[39] Bowers J.E., Dangl G.S., Vignani R., Meredith C.P. (1996). Isolation and characterization of new polymorphic simple sequence repeat loci in grape (*Vitis vinifera* L.). Genome.

[40] Bowers J.E., Dangl G.S., Meredith C.P. (1999). Development and characterization of additional microsatellite DNA markers for grape. Am. J. Enol. Vitic..

[41] Thomas M.R., Scott N.S. (1993). Microsatellite repeats in grapevine reveal DNA polymorphisms when analysed as sequence-tagged sites (STSs). Theor. Appl. Genet..

[42] Peakall R., Smouse P.E. (2012). GenAlEx 6.5: Genetic analysis in Excel. Population genetic software for teaching and research—An update. Bioinformatics.

[43] Tamura K., Stecher G., Peterson D., Filipski A., Kumar S. (2013). MEGA6: Molecular Evolutionary Genetics Analysis Version 6.0. Mol. Biol. Evol..

[44] Pritchard J.K., Stephens M., Donnelly P. (2000). Inference of population structure using multilocus genotype data. Genetics.

[45] Evanno G., Regnaut S., Goudet J. (2005). Detecting the number of clusters of individuals using the software structure: A simulation study. Mol. Ecol..

[46] Earl D.A., Vonholdt B.M. (2012). STRUCTURE HARVESTER: A website and program for visualizing STRUCTURE output and implementing the Evanno method. Conserv. Genet. Resour..

[47] Ramasamy R.K., Ramasamy S., Bindroo B.B., Naik V.G. (2014). STRUCTURE PLOT: A program for drawing elegant STRUCTURE bar plots in user friendly interface. SpringerPlus.

[48] Bitz L., Zinelabidine L.H., Rühl E.H., Bitz O. (2015). Microsatellite analysis of traditional eastern grapevine varieties and wild accessions from Geisenheim collection in Germany. Vitis.

[49] Myles S., Boyko A.R., Owens C.L., Brown P.J., Grassi F., Aradhya M.K., Prins B., Reynolds A., Chia J.-M., Ware D. (2011). Genetic structure and domestication history of the grape. Proc. Natl. Acad. Sci. USA.

[50] Aradhya M.K., Dangl G.S., Prins B.H., Boursiquot J.M., Walker M.A., Meredith C.P., Simon C.J. (2003). Genetic structure and differentiation in cultivated grape, *Vitis vinifera* L. Genet. Res..

[51] Miller A.J., Gross B.L. (2011). From forest to the field: Perennial fruit crop domestication. Am. J. Bot..

[52] Ramos-Madrigal J., Wiborg Runge A.K., Bouby L., Lacombe T., Samaniego Castruita J.A., Adam-Blondon A.F., Figueiral I., Hallavant C., Martinez-Zapater J.M., Schaal C. (2019). Palaeogenomic insights into the origins of French grapevine diversity. Nat. Plants.

[53] Mercati F., De Lorenzis G., Brancadoro L., Lupini A., Abenavoli M.R., Barbagallo M.G., Di Lorenzo R., Scienza A., Sunseri F. (2016). High-throughput 18K SNP array to assess genetic variability of the main grapevine cultivars from Sicily. Tree Genet. Genomes.

