# Supplementary material for: Genetic Structure and Relationships among Wild and Cultivated Grapevines from Central Europe and Part of the Western Balkan Peninsula
Source: Genes (Basel). 2020 Aug 20;11(9):962. doi: 10.3390/genes11090962 (PMC7563143; doi:10.3390/genes11090962)
Supplement: Supplementary file 1 [file genes-11-00962-s001.zip › supplementary/Figure_S1.docx]

| 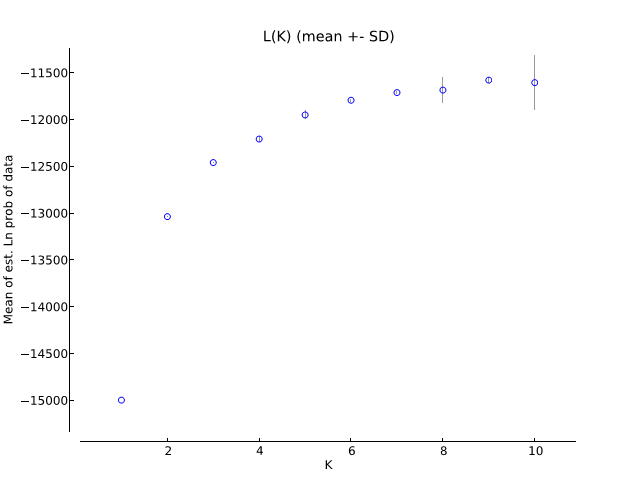  (**a**) | 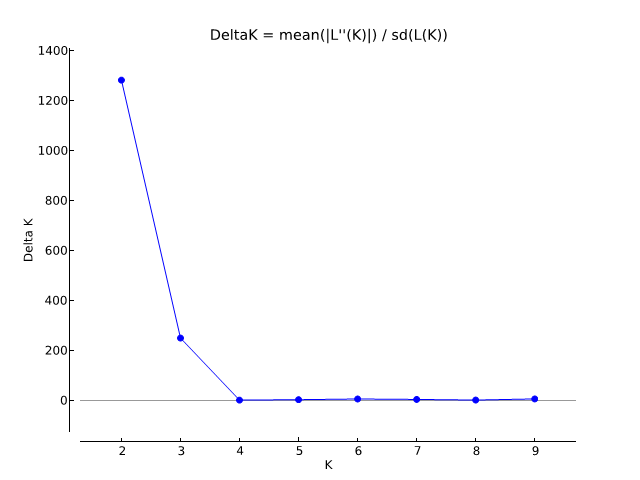  (**b**) |
| --- | --- |

**Figure** **S1.** Bayesian assignment analysis as: (**a**) a plot of mean likelihood L(K) and variance per K value exploited by STRUCTURE software on a data set of 243 individuals based on 20 SSR loci; (**b**) The optimal number of K that best fit the data set is two.
